# Using the CRISPR/Cas9 system to understand neuropeptide biology and regulation

**DOI:** 10.1016/j.npep.2016.11.010

**Published:** 2017-08

**Authors:** Elizabeth A. Hay, Christopher Knowles, Andreas Kolb, Alasdair MacKenzie

**Affiliations:** University of Aberdeen, Scotland

**Keywords:** Genome editing, Non-homologous end-joining, Homology-directed repair, Off-target effects

## Abstract

Neuropeptides and their receptors play a role in physiological responses such as appetite, stress and inflammatory pain. With neuropeptides having such diverse and important physiological roles, knocking-out the genes encoding them, their receptors, parts of their regulatory sequences, or reproducing disease associated polymorphic variants are important steps in studying neuropeptides and how they may contribute to disease. Previously, knock-outs were generated using methods such as targeted homologous recombination in embryonic stem cells but this method is costly and time-consuming. The CRISPR/Cas9 system has rapidly taken over the genome editing field and will advance our understanding of neuropeptide genes and their regulation. With CRISPR/Cas9 technology, the time and costs involved in producing transgenic animal models, is greatly reduced. In this review, we describe how the system can be used to manipulate genomic sequences by “knock-out” or “knock-in” mutations in cell lines or in animal models. We also discuss the specificity of the system and methods to limit off-target effects. When combined with the availability of genome sequences, CRISPR/Cas9 directed genome editing in vitro and in vivo, promises to provide a deeper understanding of the biology of the neuropeptides in health and disease than has ever been available before.

## Highlights

• The CRISPR/Cas9 system is set to advance our understanding of neuropeptide genes.
• The CRISPR/Cas9 system allows for efficient genome editing.
• Knock-out mutations can be produced more efficiently than knock-in mutations.
• Off-target effects of CRISPR/Cas9 can be controlled using different strategies.

## 1 Introduction

Neuropeptides play a critical role in modulating a number of physiologies, including inflammation, appetite, mood and the reward system (Pinter et al., 2013, Lang et al., 2015, Picciotto et al., 2009) that are important in maintaining the homeostatic mechanisms required for health. However, because few, if any, disease associated polymorphic variants have been found in the coding regions of neuropeptide genes using complex genome wide association studies (GWAS), interest in the biology of neuropeptides in diseases such as obesity, depression, chronic inflammatory pain and addiction has waned over the past 10 years. Critically, one important aspect of the biology of neuropeptides has not been widely explored; namely the mechanisms that maintain the correct cell specific and inducible expression of the genes that encode neuropeptides. Understanding these genomic mechanisms, and how they may be influenced by polymorphic variation or epigenetically modulated DNA-methylation, will be the key to understanding the true role of neuropeptides in health and disease. Gaining a full understanding of neuropeptide biology and the regulation of neuropeptides has been prevented in the past by an inability to easily manipulate the coding regions or regulatory sequences involved in controlling their function and expression. For example, traditional gene targeting approaches using positive-negative selection in embryonic stem cells proved to be too expensive, time consuming (Skarnes, 2015) and wasteful of animals to warrant its use in the deletion of gene regulatory elements. Thus, most of the biology of these elements, their roles in cell specific regulation of neuropeptides or the effects of polymorphic variation on their activity remains unknown. Thankfully, this situation has recently changed with the advent of genome editing technologies, the foremost of which is the CRISPR/Cas9 system.

It is fair to say that, thanks to its speed, cost and its hugely reduced animal use, the CRISPR/Cas9 system is on target for revolutionising biology. CRISPR technology has overtaken other programmable nucleases such as zinc finger nucleases and TALE proteins in terms of efficiency and specificity and has rendered traditional embryonic stem cell (ES) gene targeting largely obsolete. The CRISPR/Cas9 system is a programmable means of inducing a targeted double strand cut in genomic DNA. CRISPR/Cas9 was derived from the natural bacterial adaptive immune system that utilizes three types of CRISPR mechanisms observed in microorganisms (Hsu et al., 2014). From the point of view of genome editing in mammals the type two system is the most useful and was used in the first experiments very recently in 2013, using Cas9 for genome engineering in cells (Cong et al., 2013, Mali et al., 2013a).

### 1.1 The discovery and development of the CRISPR/Cas9 system for genome editing in eukaryotes

The genome of the adaptive bacterial immune system consists of CRISPR (clustered regularly interspaced short palindromic repeats) arrays that consist of direct repeat DNA sequences (Wiedenheft et al., 2012), interspaced with ‘spacer’ sequences that are present in known invading viruses (Sorek et al., 2008, Bolotin et al., 2005). These spacer sequences are acquired during infection by the virus and act as a primitive memory that allows the bacterium to fight the virus in future. The direct repeat sequences allow the cell to distinguish between self and non-self (Hsu et al., 2014). CRISPR based immunity is acquired through the integration of spacer segments of foreign DNA into the CRISPR locus. The CRISPR locus can then process the foreign DNA into CRISPR RNA (crRNA).

Transcribed crRNA contains the unique crRNA sequence and an adjacent palindromic repeat sequence, recognisable by a trans-activating CRISPR RNA (tracrRNA) which stimulates the processing of the RNA by RNase III (Deltcheva et al., 2011). Annealing of processed crRNA transcripts with tracrRNA allows for association with the Cas nuclease. The crRNA and tracrRNA complex guide the Cas9 enzyme to the target sequence, where it will create a double strand break in the DNA (Jinek et al., 2012).

The target DNA must contain a protospacer adjacent motif (PAM) sequence at the 3′ end of the target sequence (Jinek et al., 2012), which is specific to the Cas9 species being used (5′-NGG-3′ PAM sequence for the *Streptococcus pyogenes* Cas9 enzyme). This is the most common type two CRISPR system, and utilizes a 20 nucleotide crRNA that must immediately precede a 3 nucleotide (NGG) PAM sequence (Chylinski et al., 2013). Both the PAM and tracrRNA sequences remain constant, and are directed to a defined locus by the 20 nucleotide crRNA sequence. Because of this, it is possible to fuse the crRNA and tracrRNA sequences into a chimeric single-guide RNA (sgRNA or gRNA) (Mali et al., 2013b, Ma et al., 2014). Addition of the site specific 20 nucleotide crRNA sequence into this guide RNA is sufficient to direct Cas9, provided that the crRNA sequence is directly upstream of the NGG PAM sequence. The development of the single-guide RNA provides an easy-to-use system for relatively quick genome editing.

The development of the CRISPR/Cas9 system for mammalian genome editing has been rapid, with the establishment of online plasmid depositories such as *Addgene* (http://www.addgene.org/) and online guide RNA design tools such as http://crispr.mit.edu/. Bioinformatics tools (Heigwer et al., 2014) allow for quick and easy targeting and experiment design. In order to engineer eukaryotic genomes a minimum of two main components are required:

1. A guide RNA composed of a scaffold sequence (tracrRNA) binding to the Cas nuclease, and a targeting sequence (crRNA) which mediates binding to the intended DNA target. The tracrRNA and the crRNA are often combined to form a single guide RNA (sgRNA). The targeting sequence within the crRNA or sgRNA can be freely chosen with only minor limitations; the target DNA must be flanked by a PAM sequence at the 3′ end (Jinek et al., 2012), and the complement of the PAM sequence should not be present on the guide RNA.
2. The Cas9 protein, mRNA or a plasmid encoding the protein. Plasmid expression constructs have been designed (e.g. pX330; Addgene #42230) that only require insertion of 20 nucleotide crRNA sequence required for directing the Cas9 complex to a defined locus. In this case, the sgRNA is expressed from the U6 polymerase III promoter, and requires the crRNA to start with a G; the only constraint to targeting specificity with this system is the GN_20_GG motif (G + crRNA + NGG PAM). The gene encoding the Cas9 protein has undergone a number of alterations, including the removal of one or both of its DNA cutting domains, and the addition of a nuclear localisation signal to guide the Cas9-gRNA complex to the nucleus.
  It is important to note, however, that the CRISPR system only permits directed cutting of the target genome and plays no further role in its subsequent repair. This aspect is governed by the host cells natural repair mechanisms. The next section of this review will explore the cellular pathways that repair CRISPR mediated genomic breaks and how they can be manipulated to produce the desired outcome.

### 1.2 Repair of CRISPR induced insertions/deletions by cellular repair mechanisms

Following a double strand break, the cells natural repair system will attempt to fix the break. There are two types of system used by mammalian cells, the first is the rapid and efficient non-homologous end joining (NHEJ) system (Valerie and Povirk, 2003). However, non-homologous end joining is error prone and has been known to introduce unwanted sequences that compromise attempts to introduce subtle genomic changes, such as introduction of disease associated allelic variants or the targeted introduction of larger sequences, such as reporter genes or antibody epitopes. In contrast, homology directed repair (HDR) is more accurate than NHEJ but is less efficient in the presence of double strand cuts.

Off target activity can be reduced by up to 1500 fold by introducing paired single strand “nicks” as opposed to double stranded cuts. This is achieved using a mutant version of the Cas9 enzyme (Cas9n) also called D10A mutant nickase (Ran et al., 2013). If nickases are introduced with DNA constructs called “repair templates” then the HDR pathway can insert sequences within the repair template by homologous recombination thus providing a method to introduce or “knock-in” novel sequences into the genome. The use of the “nickase” is also thought to reduce possible off-target effects whereby the Cas9 cuts sequences elsewhere in the genome that only differ from the target sequence by one or two base pairs. Using a combination of nickases and repair templates, targeted insertions, previously known as “knock-ins” when performed using ES-cell targeting, can be achieved.

## 2 Use of CRISPR/Cas9 technology for genome editing in transformed/immortalised cell culture

One of the most widespread uses of the CRISPR/Cas9 system in eukaryotes has been its use in transformed cell lines (Chu et al., 2015). These immortalised cell types are easy to grow and transfect and many of them take on morphological features characteristic of neurones. These cell types include SH-SY5Y neuroblastoma cells that, in the presence of morphogens such as retinoic acid, produce structures that resemble axons/dendrites and express many neurone specific proteins (Korecka et al., 2013). Editing the genomes of these cells involves the transfection of Cas9-sgRNA expression plasmids (like pX330) that express both the guide RNA and the corresponding Cas9 nuclease.

Homology-directed repair has a low efficiency in mammalian cells and non-homology directed repair is predominant (He et al., 2016). NHEJ directed integrations occur at a 1000 times greater frequency than HDR (Vasquez et al., 2001). In fact, recent research has shown that targeted knock-in is more efficient in mammalian cells through the NHEJ process compared to the HDR process (He et al., 2016). The study also suggests that precise ligations following NHEJ repair can be produced through the conventional NHEJ mechanism.

Homology-directed repair may be enhanced through inactivating molecules in the non-homologous end-joining repair pathway to increase the likelihood of the homology-directed repair pathway being initiated (Chu et al., 2015). However, the method used to enhance HDR should be carefully considered depending on the context of the experiment, as the NHEJ pathway is important for repairing DNA damage in cells. The efficiency of HDR is dependent on the targeted gene locus. 36% gene targeting efficiency was observed at the AAVS1 locus in HEK293 cells when non homologous end joining was suppressed (Chu et al., 2015). In contrast, the homologous recombination efficiency at the TERT locus in HEK293 cells (chromosome 5p15. 33) was much lower (< 1%) (Xi et al., 2015). At least two guide RNA sequences were used in both of these reports with varying efficacy. This supports similar observations that both the target site, and the guide RNA sequence used to target it have drastic effects on targeting efficiency (Doench et al., 2014).

Illegitimate recombination via non-homologous end joining (NHEJ) is more efficient than HDR at repairing double strand breaks in mammalian cells (Lieber, 2010). The introduction of targeted double strand breaks at a locus surrounded by homology from a repair template can induce HDR by up to 100 fold, but an increase in illegitimate recombination by up to 1000 fold occurs in tandem (Sargent et al., 1997). Even with selection strategies specific for HDR events in somatic cells, without double strand break induction, the ratio of homologous/illegitimate recombination frequencies can be as low as 1:4000 (Adair et al., 1989).

Increased frequencies of homologous recombination in stem cell lines (Tichy et al., 2010) is a result of elevated proteins known to be involved in homologous recombination (Matsunami et al., 2008). Stem cells show decreased expression of DNA ligase IV, which is a rate limiting factor for NHEJ (Riballo et al., 2009) in the majority of mammalian cells.

Suppression of DNA ligase IV in mammalian cells has been achieved with siRNA knockdown, a small-molecule inhibitor (SCR7) and adenoviral proteins (E1B55K and E4orf6) that mediate its degradation (Chu et al., 2015). Chu et al. (2015) demonstrate that NHEJ suppression in HEK293 cells can increase HDR efficiency by five to seven fold (5% without NHEJ suppression to 36% with it). However, these efficiencies were reported at the permissive (AAVS1) locus using a ‘traffic light reporter’ assay described by (Certo et al., 2011). Similar experiments using the same reporter system in HEK293 cells with siRNA knockdown achieved HDR efficiencies of 4%, with NHEJ efficiency remaining high (25–30%) (Certo et al., 2011). The authors attribute such variability to differences in targeting and screening strategies to score genome manipulation events. With this in mind, neither article provided selection pressure to augment these rates. Chu et al. (2015) did note a 100% efficiency of targeting a puromycin marker into murine stem cells via HDR and selection. Augmented HDR at endogenous loci with NHEJ suppression in porcine somatic cells (fetal fibroblasts) can reach efficiencies of 44% (Park et al., 2016).

NHEJ can also be suppressed using reversible inhibitors of cell cycle progression to temporarily lock cells within the G2/M phase (Yang et al., 2016). This method shows no detrimental effects or toxicity in human stem cells, and increases CRISPR mediated HDR efficiency by up to 6 fold. Additional chemical inhibition of NHEJ via SCR7 showed no further increase in HDR efficiencies. This indicates that cell cycle arrest and NHEJ inhibition share similar mechanisms. Zero toxicity was also observed when restricting HEK293 cells to the late G2 phase with a reversible chemical inhibitor (Nocodazole), which improved CRISPR mediated HDR efficiency by 11% (Lin et al., 2014). Both of these reports note a preference for this method over chemical inhibition of NHEJ for increased reliability, reduced cost and ease of implementation.

The asymmetric manner in which Cas9 binds to, and cleaves DNA can also be used to increase HDR efficiency. Dissociation of Cas9 from cleaved DNA primarily exposes the non-target 3′ strand (Richardson et al., 2016). Single stranded oligodeoxynucleotides with homology to this exposed region show increased HDR efficiencies by up to 60% in HEK293 cells. The efficiency of homology-directed repair can be enhanced by optimising the design of single-stranded DNA donors to target the 3′ strand which is released first.

## 3 Producing knock-out mutations in mice using CRISPR/Cas9

**Fig. 1:**
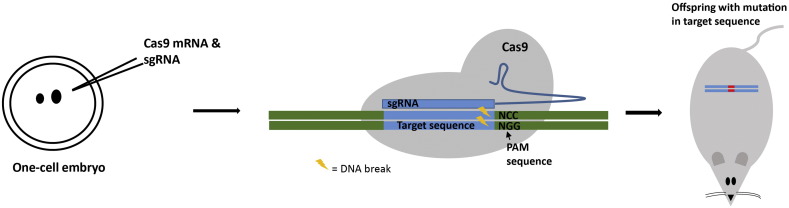
Producing a knock-out mutation. The production of mice with a knock-out mutation produced through the non-homology end-joining (NHEJ) repair mechanism. One-cell embryos, isolated from a donor mouse, are microinjected with Cas9 mRNA and single guide RNA (sgRNA) specific to the target DNA. The mechanism of the system is illustrated, with the sgRNA binding to the target genomic DNA (blue region), guiding the Cas9 enzyme (in grey) to the region which then produces the double strand break. The double strand break is repaired by NHEJ, which could include insertions, deletions or frameshift mutations, resulting in gene disruption. Embryos are transferred to a pseudopregnant host mouse and offspring are genotyped to test for the presence of the knock-out mutation.

Although useful for understanding the biology of neuropeptides in a single cell context the use of CRISPR technology in transformed cells has limited use in understanding the regulatory systems that control the cell specific and inducible expression of neuropeptide genes in vivo. In the presence of one guide RNA, the CRISPR/Cas9 system can be used to induce mutations via insertion or deletion of genetic information following NHEJ mediated repair at the Cas9 cleavage site. Such mutations at specific genes or gene regulatory regions will disrupt expression (Fig. 1). The precision of this method is limited by the unpredictability of NHEJ mediated repair junctions.

An alternative method, which can be used to delete larger sequences, would be to use two single-guide RNA sequences. These should flank the sequence to be deleted. Each guide RNA would direct the Cas9 to produce a double strand break at their target genomic sequence. If the two double-strand breaks occur simultaneously, the DNA sequence that they flank will be cut-out and the natural-repair system will join the two ends of DNA together.

Many have concerns over the potential off-target effects of these methods as mismatches can occur between the target and the 20 base-pair guide RNA. One method to reduce off-target effects is to use a Cas9 nickase mutant along with two off-set guide RNAs on opposite strands of the target DNA (Ran et al., 2013). A mutation in a catalytic residue in one of the two nuclease domains of Cas9 means that only one nuclease will cut, so a single strand cut in the template DNA is produced instead of a double strand break. If two single strand breaks are produced on opposite but proximal strands, this will allow for a double strand break to be produced. When compared to off-target mutations produced by the Cas9 nuclease, it has been suggested that the double-nicking method may reduce the risk of off-target effects (Ran et al., 2013).

Conditional knock out mouse lines can also be developed using CRISPR/Cas9 to introduce recombinase target sites (RTS). By using two guide RNAs to target the flanking ends of a gene and insert a set of RTS, the genomic information in-between these RTS then becomes excisable after activation of a recombinase enzyme (Yang et al., 2013). Using CRISPR/Cas9 to augment this process is considerably faster than the traditional targeting methods in embryonic stem cells (Capecchi, 2005).

### 3.1 Addressing the problem of “off-target” effects

There has been much concern raised in the scientific community over the specificity of the CRISPR/Cas9 system. Many have become concerned about “off-target” effects, where the guide RNA, in addition to cutting the target site, would guide the Cas9 enzyme to other almost homologous sites. New techniques, however, have been developed to limit “off-target” effects, such as mutations in the Cas9 gene that produce a nicking enzyme or an enhanced specificity enzyme. 1500 fold reductions in off target specificity (Ran et al., 2013) can be achieved with paired targeting of a Cas9 nickase mutant (Cas9n) (Mali et al., 2013a). Reduced off target specificity comes at the expense of HDR efficiencies, with frequencies as low as 5% in human stem cells being demonstrated (Rong et al., 2014).

The methods for the delivery of Cas9 have significant effect on targeting efficiency. Cas9-sgRNA ribonucleoprotein complexes can be directly transfected into mammalian cells (Zuris et al., 2015). This method provides significant improvements in DSB induction with minimised toxicity compared to plasmid transfection. Zuris et al. (2015) also provide methods for recombinant production of Cas9, in vitro transcription of sgRNA and ribonucleoprotein complex formation.

Other methods have also been developed to attempt to overcome the problem of potential off-target effects. For example, an enhanced specificity *Streptococcus pyogenes* Cas9 (eSpCas9) variant has been shown to reduce off-target mutations (Slaymaker et al., 2015). The research team neutralised the positively charged non-target strand groove in SpCas9 by changing amino acids. The groove is thought to play a role in stabilising the non-target strand of DNA and therefore, by neutralising this groove its interaction with the non-target strand would become less stable, and in turn, strong base-pairing of the guide RNA at the target would be needed.

An alternative approach to reducing off target effects is explored in a study by Hay et al. (2016) in this issue, who microinjected gRNA and Cas9 mRNA into the cytoplasm of one cell mouse embryos (demonstrated in Fig. 1). This method succeeded in producing a high proportion of mice in which both copies of enhancers of the galanin gene and the gene encoding cannabinoid receptor-1 had been deleted. Intriguingly, on closer analysis Hay et al. (2016) were able to demonstrate a complete lack of detectible off-target effects, in regions of the genome predicted to act as potential off target sites, in these successfully targeted mouse lines. They concluded that many of the off-target effect problems previously reported for the CRISPR system may have stemmed from studies where plasmids expressing high levels of Cas9 and gRNA were transfected into actively dividing immortalised cancer derived cell lines. Due to high levels of Cas9 and gRNA in these cells, it was not surprising that off-target effects eventually occurred. However, the gRNA and Cas9 mRNA injected into one-cell embryos allowed sufficient time for site specific targeting but, because of rapid degradation, did not persist long enough to induce off target effects. Additionally, it has been shown that following the introduction of DNA to the pronucleus (circular plasmid DNA), a significantly reduced developmental efficiency of the embryos was observed, compared to cytoplasmic, RNA injection (Horii et al., 2014). Therefore, cytoplasmic microinjection of RNA is likely to have contributed to the high survival rate and to specific targeting in the study by Hay et al. (2016).

### 3.2 CRISPR interference

An alternative to producing knock-outs or mutations in the genome is the use of the CRISPR interference (CRISPRi) method. This method sterically represses transcription and works in the same way as the knock-out method, where a sgRNA allows a Cas9 protein to target a specific sequence. However, in this system, the Cas9 protein is mutated so that it is catalytically inactive (dCas9) and binds to the target sequence to block transcriptional apparatus at that site (Larson et al., 2013). Therefore, gene transcription is inhibited, and the activity of the gene is removed without cutting or mutating the DNA sequence. This gene knock-down can be reversed (Qi et al., 2013) as there is no change to the DNA sequence. The level of gene repression can also be modulated by using the CRISPRi system, by using multiple sgRNAs to target parts of the same gene (Larson et al., 2013) or multiple sgRNAs can be used to concurrently control several different genes, whilst maintaining specificity (Qi et al., 2013). CRISPRi is therefore a valuable tool which can be used for and has the potential for development in several applications including screening for gene function and for the production of inducible gene expression systems (Dominguez et al., 2016).

## 4 Using CRISPR/Cas9 to introduce single base pair changes

**Fig. 2:**
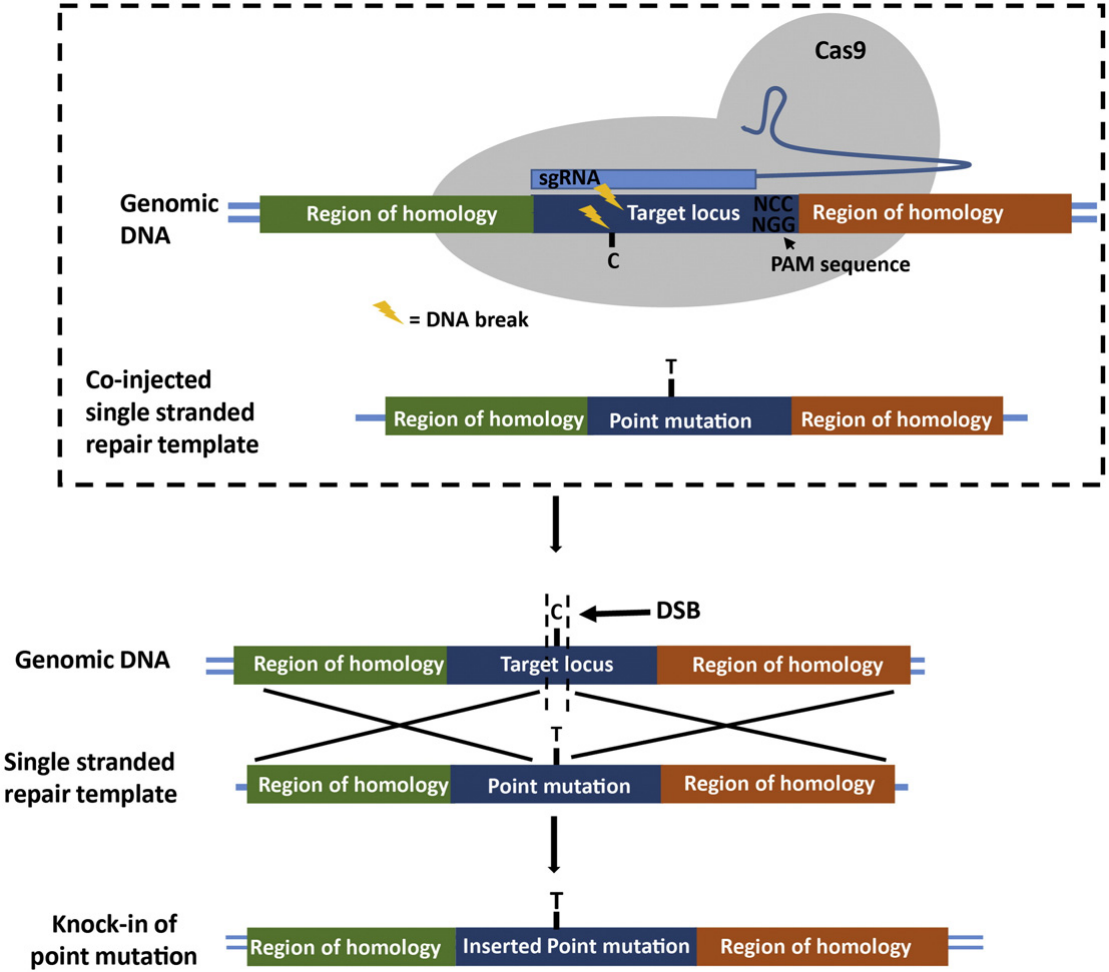
The introduction of a point mutation. A single stranded repair template is co-injected with the sgRNA and the Cas9 mRNA. This single stranded oligonucleotide contains a point mutation flanked by homology arms (indicated by green and orange regions of homology) to elicit homology directed repair. After Cas9 produces a double strand break (DSB), homology directed repair follows, with the insertion of the point mutation into the genomic DNA target region. In this example, a C to T point mutation is produced and demonstrates how different alleles of a SNP can be studied using CRISPR.

The production of genetically modified animals is important for the study of the development of disease and their treatment. Whilst tissue or primary cell culture provide useful models for researching disease, results from in vivo models often differ because of the genetic and physiological make-up of a complete organism (Doyle et al., 2012). Although tissue and primary cell culture models are relevant for the study of disease, in vivo studies provide more relevance to human disease. Therefore, the knock-in of specific DNA sequences such as, sequences containing SNPs, conditional alleles or even human versions of genes or cis-regulatory regions is critical for understanding disease progression and treatment. Traditionally, DNA sequences have been introduced to mice via artificial chromosomes, viral vectors or by the pronuclear microinjection of embryos with DNA which randomly integrates into the genome or homologous recombination in embryonic stem cells and more recently, the use of ZFNs (Brown et al., 2013). However, the CRISPR/Cas9 system provides a far more efficient, rapid method for the introduction of DNA sequences in mice. By co-injecting Cas9 and a sgRNA specific to the target sequence along with a single stranded oligonucleotide template DNA strand which has homology to the target site at either end, homology-directed repair is initiated, resulting in insertion of the DNA strand. This method could be used for example, to insert specific base pair mutations to enable the study of SNPs (Inui et al., 2014, Armstrong et al., 2016) (Fig. 2).

## 5 Introducing larger DNA constructs and reporter constructs

**Fig. 3:**
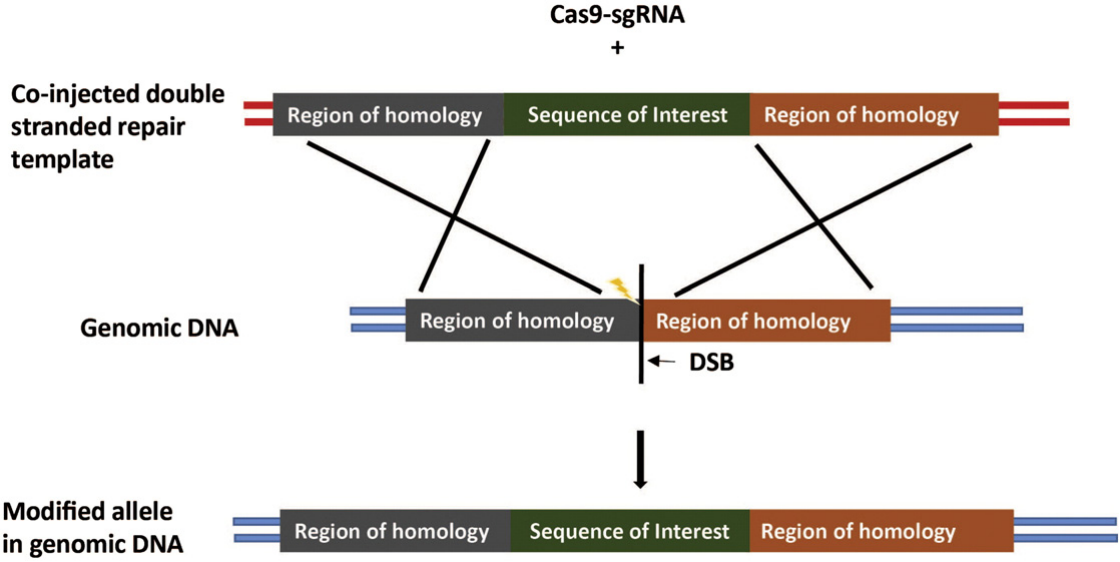
The introduction of a large DNA construct. Larger DNA constructs follow similar principals to single base knock-ins, except that a double stranded DNA construct is usually used as the repair template. In this example, the double stranded repair template contains a sequence of interest that is to be inserted into the genome, which is flanked by two homology regions that correspond to the target locus. One of these homology arms should be no less than 100 bp away from the intended sgRNA cleavage site. Whilst the transgene size can vary, homology regions are typically between 800–1400 kbp.

Although homology-directed repair is not highly efficient, the knock-in of large DNA constructs has been achieved. Typically, a double stranded DNA repair template is used for the integration of large constructs via HDR (Lee et al., 2015). An example of the knock-in of a larger construct is shown in Fig. 3. HDR efficiency is rapidly lost with increasing insert size, with particular reference to those over 3 kbp (Perez et al., 2005). Interestingly, several studies (prior to CRISPR) showing high efficiency of HDR with inserts > 1 kb (Liang et al., 1998, Brenneman et al., 1996), direct double strand breaks to sites that are included within a double stranded DNA repair template. Resection of these broken ends provides immediate single stranded homology to the genomic locus, and annealing would induce HDR via routes similar to that seen with single stranded oligodeoxynucleotides. Single strand oligonucleotide repair templates can be used for HDR of 100 bp inserts as Park et al. (2016) have demonstrated. However, their efficiency at stimulating HDR is up to 8 fold less than that of an identical double stranded plasmid donor (Davis and Maizels, 2014). This may be a reflection of their limited transfection efficiency and stability. The use of single-stranded oligodeoxynucleotides (ssODNs) with the CRISPR-Cas9 system has allowed for the knock-in of GFP in rat and even the knock-in of a 200 kb BAC containing a human gene or 6.2 kb human gene in rat zygotes (Yoshimi et al., 2016). Long ssODNs can be used as the target donor and co-injected into the embryo pronucleus with a gRNA targeting the genomic DNA and Cas9 mRNA. For example, Yoshimi et al. (2016) knocked in a GFP reporter Cassette into rat zygotes using an lsODN of 837 bp with 60 bp homologous arms at either end. Alternatively, two ssODNs can be co-injected with plasmid DNA, gRNAs (to target the cut site of the genomic DNA and to target the plasmid DNA cut site) and Cas9 mRNA. The two ssODNs will ligate the ends of the plasmid DNA into the genomic DNA. This method has been used by the same group to knock-out a gene cluster and knock-in one human gene into rats. The method allows for a more simplistic approach to the knock-in of DNA without the need to construct homology arms.

Another group however, has successfully knocked-in 8 or 11 kb sequences into the genomic ‘safe harbour’ site, Rosa26, using homology directed repair in C57BL/6 zygotes (Chu et al., 2016). Interestingly, they also indicate that knock-in frequency could be increased when Cas9 mRNA was combined with Cas9 protein and microinjected into zygotes which were cultured to blastocysts. They suggest that the activity of the Cas9 is more persistent as the microinjected protein would first initiate double strand breaks and homology-directed repair, and this initial activity is enhanced as new Cas9 is translated from mRNA.

## 6 Conclusions

The advent of the CRISPR/Cas9 system has greatly enhanced the efficiency of genome editing. Its use in cells and animal models has the potential for understanding neuropeptide gene function and gene regulation by targeting both genes and their regulatory elements. Therefore, the technology is likely to have a significant role in elucidating gene regulation of neuropeptides leading to a better understanding of the physiological roles and disease susceptibility associated with mis-regulation of neuropeptides.

In this review, we have summarised different mechanisms of the CRISPR/Cas9 system, highlighting the fact that producing a knock-out mutation can be done more efficiently than producing a knock-in mutation. The efficiency of knock-in may be further reduced by that fact that the introduction of DNA to the pronucleus can be damaging, due to physical damage from the microinjection and by possible random integration of the DNA into the genome. Another possible problem with introducing DNA to one-cell stage embryos is that DNA is known to adhere to glass, and therefore, less DNA than expected may be delivered to the cell via the glass micropipette.

We have also highlighted methods used to limit off-target effects, such as the double nicking method and by the introduction of RNA to one-cell stage embryos rather than DNA. Furthermore, the concept of inducible and reversible gene knock-out by the CRISPR interference method has been introduced. Overall, the CRISPR/Cas9 technology has been quite extensively examined, is developing rapidly and has great potential for exploring gene regulation of neuropeptides.
